# Migration Status, Work Conditions and Health Utilization of Female Sex Workers in Three South African Cities

**DOI:** 10.1007/s10903-012-9758-4

**Published:** 2012-12-13

**Authors:** Marlise Richter, Matthew F. Chersich, Jo Vearey, Benn Sartorius, Marleen Temmerman, Stanley Luchters

**Affiliations:** 1 Department of Obstetrics and Gynaecology, International Centre for Reproductive Health, Ghent University, Ghent, Belgium; 2 African Centre for Migration & Society, University of the Witwatersrand, Johannesburg, South Africa; 3 Faculty of Health Sciences, Centre for Health Policy, School of Public Health, University of the Witwatersrand, Johannesburg, South Africa; 4 School of Public Health, Faculty of Health Sciences, University of the Witwatersrand, Johannesburg, South Africa; 5 Centre for International Health, Burnet Institute, Melbourne, Australia; 6 School of Public Health and Preventive Medicine, Monash University, Melbourne, Australia

**Keywords:** Sex work, Condoms, Health care utilization, Migration status, South Africa

## Abstract

Intersections between migration and sex work are underexplored in southern Africa, a region with high internal and cross-border population mobility, and HIV prevalence. Sex work often constitutes an important livelihood activity for migrant women. In 2010, sex workers trained as interviewers conducted cross-sectional surveys with 1,653 female sex workers in Johannesburg (Hillbrow and Sandton), Rustenburg and Cape Town. Most (85.3 %) sex workers were migrants (1396/1636): 39.0 % (638/1636) internal and 46.3 % (758/1636) cross-border. Cross-border migrants had higher education levels, predominately worked part-time, mainly at indoor venues, and earned more per client than other groups. They, however, had 41 % lower health service contact (adjusted odds ratio = 0.59; 95 % confidence interval = 0.40–0.86) and less frequent condom use than non-migrants. Police interaction was similar. Cross-border migrants appear more tenacious in certain aspects of sex work, but require increased health service contact. Migrant-sensitive, sex work-specific health care and health education are needed.

## Background

Southern Africa is home to the largest population of people with HIV globally [56]. A meta-analysis showed that sex workers in sub-Saharan Africa were 12.4 times more likely than the general population to acquire HIV, with 95 % confidence interval (CI) estimates ranging from 8.9 to 17.2 [6]. Further, female sex workers (FSWs) who are migrants in lower-income countries have higher HIV risks than non-migrants [37] Despite this, appropriate legal, policy and programmatic responses to HIV, migration and sex work are lacking in Africa [42, 44, 48, 57, 64] and sex work remains mostly criminalised across the continent [50, 64].

Internationally, studies have highlighted clear linkages between migration and sex work [2, 10, 11, 58]. In southern Africa, whilst several studies have documented associations between migration and informal livelihood activities [1, 27, 36, 38, 64], little research has focused specifically on the overlap between sex work and migration.

**Fig. 1:**
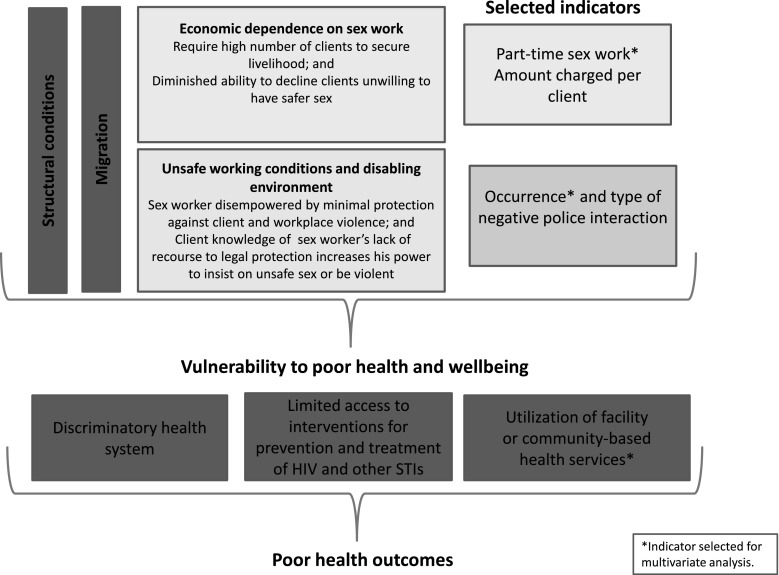
Factors influencing health outcomes of non-migrant, internal migrant and cross-border migrant sex workers

This study therefore assessed selected structural determinants of vulnerability of migrant FSWs (economic environment and working conditions) and whether access to health services varies between non-migrants, internal migrants and cross-border migrants. The study, in four sites in South Africa, evaluates outcomes based on a conceptual framework (Fig. 1). This framework draws on previous evidence showing that health status and HIV risk among sex workers is contingent on sole economic dependence on sex work, safety of the work environment and degree of responsiveness of health services [8, 13, 40, 46, 67]. Clients often demand unprotected sex [12, 35, 39], and the ability of sex workers to negotiate safer sex depends on their degree of economic vulnerability, and the prevailing power relations between sex workers and clients, and between sex workers and law enforcement agencies [7, 16, 67]. In South Africa, cross-border migrants face high levels of police harassment [25] and difficulties in accessing health services because of language problems or xenophobic health care workers [23]. We hypothesise that these experiences extend to migrant sex workers, and influence their economic dependence on sex work, safety of work conditions and contact with health services.

## Methods

### Study Setting

Cross-sectional surveys with self-identified FSWs were conducted around the time of the 2010 Soccer World Cup, during which few changes in FSW demographics were documented [43]. Two contrasting areas of Johannesburg, the largest city in South Africa, were selected: Hillbrow and Sandton. The inner-city area of Hillbrow was chosen as it has a well-known, long-standing sex trade and is a popular destination for newly-arrived migrants [32, 33, 42, 51, 62, 69]. Sandton, by contrast, is a wealthy suburb and business district [5] with a visible outdoor sex industry. The Rustenburg site, in a predominantly rural province, comprised informal settlements within a platinum mine area about 15 km outside the city. Its sex work industry mainly serves the local mining community [4]. The coastal city of Cape Town is a popular international tourist destination [45], with a relatively well documented sex work industry [17–20, 35]. Commercial sex work, for purposes of this study, was defined as the exchange of sexual services for financial reward in women above 18 years. Detailed methods are described elsewhere [43].

### Data Collection

Between May and September 2010, university-based researchers collaborated with two non-governmental organisations—the Sex Worker Education and Advocacy Taskforce (SWEAT) and Sisonke Sex Worker Movement [68]. Sex worker peer educators and other sex workers attended a training workshop addressing research ethics, participant selection and interviewing. Ten research assistants were selected per site, with those in Hillbrow also collecting data in Sandton.

Research assistants administered a 43-item semi-structured questionnaire to approximately 60 sex workers each. To minimise selection bias, they approached every third woman known to them as a sex worker and invited her to participate. Questionnaires were adapted from tools used in previous studies with sex workers in Mombasa, Kenya [29] and research on migration and access to health care in Johannesburg [59]. Study tools were translated from English into Afrikaans, isiXhosa, isiZulu and Setswana.

### Ethical Considerations

Participants provided written informed consent and were offered a cell-phone airtime or grocery voucher of 20 South African Rand (~US$3) for time taken in interview. Women were referred to local counselling, health and legal assistance organizations, when required. Participants were given female condoms and information about a newly established toll-free sex worker helpline. As sex work is criminalized in South Africa [9], no identifying information was collected. Study databases were password-protected, with access restricted to the research team. The University of the Witwatersrand Human Research Ethics Committee approved the protocol (Protocol no. H100304).

### Study Measures & Data Analysis

Data were entered in duplicate in Microsoft Access by separate data clerks. Participants were asked to indicate if they had been interviewed previously and data from repeat interviews (356 of 1,696 women) was excluded from analysis. We compared socio-demographic characteristics and study outcomes between three study groups: (1) non-migrant females working in the province of their birth, (2) internal migrants, born in different province from where they work, and (3) cross-border migrants, women born in another country.

Based on previous evidence, three categories of risk factors were defined, each measured as a binary outcome: economic dependence on sex work [8] (earns income outside sex work, i.e. part-time sex workers), unsafe work environment [7] (had negative interaction with law enforcement in past year) and health services contact [8] (contact in past month with facility- or community-based health services such as peer education or outreach). Part-time sex work was defined as having any other income aside from sex work [22]. Free text descriptions about contact with the police in the preceding year were coded as a “negative interaction” if it concerned police violence, arrest, harassment, theft, bribery or fines. Conversely, “positive interaction” denoted police assistance with, for example, laying a complaint or warning a participant about potential danger. Weekly income was calculated from the mean amount charged with last two clients, and multiplying that by the number of clients in past week (7.5 South African Rand = 1 US Dollar).

Chi square tests were used to detect differences between categorical variables. For continuous variables, The Kruskal–Wallis test compared those with a non-normal distribution, and ANOVA test those with a normal distribution. Bivariate analysis was conducted to assess possible confounding by site. Associations between migration group and the three study outcomes were assessed in multivariable logistic regression analysis, controlling for site of enrolment, socio-demographic and sex work confounders. Variables associated with the primary outcome in bivariate analysis or in similar studies were included in the initial model and retained if their removal from the model markedly altered the model fit.

## Results

### Population Description

Of 1,653 participants, 17 did not state birthplace and were excluded from analysis, while 240 (14.7 %) were non-migrants, 638 (39.0 %) internal migrants and 758 (46.3 %) cross-border migrants. Participants were a mean 29.7 years, similar in the three study groups. Across groups, more than 40 % of participants had spent five or more years in sex work. There was a difference in number of dependents (child and adult) between the groups: a median two for non-migrants, three for internal migrants and four for cross-border migrants (*P* < 0.001). More cross-border migrants (39.6 %) had a regular partner than internal migrants (30.6 %; *P* < 0.001) or non-migrants (27.9 %; *P* = 0.01). However, cross-border migrants who had a regular partner were less likely to live with him/her (34.1 %) than their internal migrant (43.2 %; *P* < 0.001) or non-migrant (54.5 %; *P* = 0.01) counterparts.

Over one-third (276/733) of cross-border migrants had completed secondary school or some tertiary training, 2.2 fold more than the other two groups (95 % CI odds ratio [OR] = 1.5–3.1). These levels were similar between internal migrants and non-migrants (OR = 1.1; 95 %Cl = 0.8–1.6). Cross-border migrants took up sex work at an older age (mean = 24.9 years, standard deviation [SD] = 5.3) than non-migrants (mean = 23.0 years; SD = 5.4; *P* < 0.001). Approximately 60 % of all migrants—similar among internal (332/551) and cross-border migrants (357/600)—started sex work within two years of arrival in the city. Notably, a quarter of cross-border participants (152/626) were sex workers before leaving their place of birth compared to only about 10 % of internal migrants (58/539, *P* < 0.001; data not shown).

**Fig. 2:**
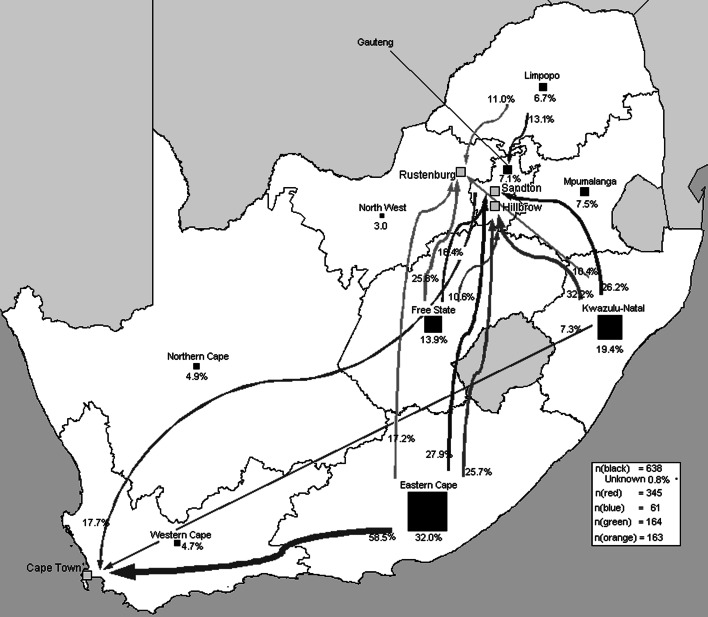
Place of origin of internal migrants according to research site

**Fig. 3:**
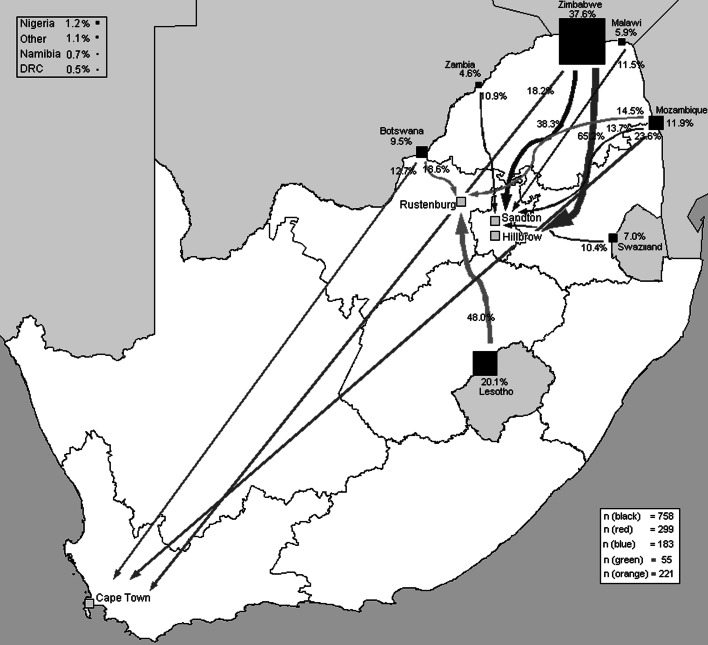
Place of origin of cross-border migrants according to research site

Eastern Cape province was the biggest contributor of internal migrants (204) to the four sites, exceeding the 124 internal migrants from KwaZulu-Natal and 89 from the Free State (Fig. 2). This echoes recent findings that the Eastern Cape is one of South Africa’s poorest provinces, with high rates of outmigration [41, 52]. Hillbrow and Sandton had the highest proportion of cross-border migrants (51.9 %, 308/594 in Hillbrow and 66.1 %, 193/292 in Sandton). For all sites, most cross-border migrants hailed from South Africa’s neighbouring countries—notably Zimbabwe (Fig. 3). Participants from Zimbabwe had a greater number of total dependants (median = 5), than South Africans (median = 3) or those born in other countries (median = 4; *P* ≤ 0.001). Half of non-migrants (117/233) and a third of internal migrants solicited outdoors (195/600; *P* < 0.001), compared to only 22.8 % of cross-border migrants (*P* < 0.001). The latter group predominately worked indoors (52.0 %, 372/715), especially in Hillbrow where two-thirds worked indoors (186/282) Table 1.

**Table 1:** Description of socio-demographics, sex work and migration characteristics of female sex workers in four sites in South Africa (N = 1636)

Variables	Non-migrant n = 240	Internal migrant n = 638	Cross-border migrant; n = 758	*P* value^b^
Age, mean years (SD)	29.6 (6.8), n = 240	29.9 (6.5), n = 633	29.7 (6.4), n = 757	<0.78^c^
Site, n/N (%)				
Hillbrow, Johannesburg	35/240 (14.6 %)	246/638 (38.6 %)	299/758 (39.5 %)	
Sandton, Johannesburg	20/240 (8.3 %)	64/638 (10.0 %)	183/758 (24.1 %)	<0.001
Cape Town	134/240 (55.8 %)	164/638 (25.7 %)	55/758 (7.3 %)	
Rustenburg	51/240 (21.3 %)	164/638 (25.7 %)	221/758 (29.2 %)	
Education, n/N (%)				
Primary incomplete	45/228 (19.7 %)	117/614 (19.1 %)	134/733 (18.3 %)	<0.001
Completed primary	133/228 (58.3 %)	350/614 (57.0 %)	323/733 (44.1 %)	
Completed secondary school	42/228 (18.4 %)	106/614 (17.3 %)	223/733 (30.4 %)	
Some tertiary training	8/228 (3.5 %)	41/614 (6.7 %)	53/733 (7.2 %)	
Median number of dependants,(IQR)	2 (1–4)	3 (2–6)	4 (2–6)	<0.001^d^
Relationship status, n/N (%)				
Single	170/237 (71.7 %)	435/626 (69.5 %)	451/747 (60.4 %)	<0.001
Regular partner	66/237 (27.9 %)	190/626 (30.6 %)	296/747 (39.6 %)	
Lives with regular partner	36/66 (54.5 %)	82/190 (43.2 %)	101/296 (34.1 %)	
Age at sex work debut, mean years (SD); n	23.0 (5.4); n = 212	24.0 (5.1); n = 585	24.9 (5.3); n = 684	<0.001^c^
Duration in sex work, n/N (%)				
<1 year	33/217 (15.2 %)	78/583 (13.4 %)	134/692 (19.4 %)	0.03
1–5 years	81/217 (37.3 %)	232/583 (39.8 %)	278/692 (40.2 %)	
>5 years	103/217 (47.5 %)	273/583 (46.8 %)	280/692 (40.5 %)	
Main place solicit clients^a^, n/N (%)				
Indoors	64/233 (27.5 %)	259/600 (43.2 %)	372/715 (52.0 %)	<0.001
Outdoors	117/233 (50.2 %)	195/600 (32.5 %)	163/715 (22.8 %)	
Combination of venues	52/233 (22.3 %)	146/600 (24.3 %)	180/715 (25.2 %)	
Sex work initiation, n/N (%)				
Before arrival in city	–	105/551 (19.1 %)	177/600 (29.5 %)	<0.001
Within 2 years of arrival in city		332/551 (60.3 %)	357/600 (59.5 %)	
2 or more years of arrival in city		114/551 (20.7 %)	66/600 (11.0 %)	
Median months since leaving birthplace, (IQR)	–	79.2 (28.2–131.2)	47.2 (18.1–111.0)	<0.001^d^
Median months since arrival in current workplace, (IQR)	–	67.7 (24.2–123.8)	41.0 (16.2–90.0)	<0.001^d^

*SD* standard deviation, *IQR* inter-quartile range

^a^Indoors includes working from brothels, bars or massage parlours; outdoors includes street-based sex workers; and women reporting both these were classified as combination venues

^b^Chi square test unless indicated

^c^ANOVA test

^d^Kruskal-Wallis test; All tests compare distribution across all three study groups apart from time since leaving birthplace and arrival in workplace

### Economic Dependence on Sex Work

More than a third (256/723) of cross-border migrants worked as part-time sex workers, in contrast to a quarter of internal migrants (150/606; *P* < 0.001; Table 2), and a fifth of non-migrants (40/213; *P* < 0.001). In bivariate analysis assessing this outcome in each site, patterns of part-time work across the study groups were similar to overall findings, except in Rustenburg. Here, for each migrant group, about 20 % worked part-time. After adjusting for confounding factors including site, cross-border migrants were 2.3 times more likely to work as part-time sex workers than non-migrants (95 % CI adjusted-OR [AOR] = 1.5–3.7; Table 3). Similarly, women with some tertiary training compared to those who had not completed primary school had a twofold odds of being a part-time sex worker (95 % CI AOR = 1.1–3.6). Women who had a permanent partner were 2.8 times more likely to be a part-time sex worker than those who were single (95 % CI AOR = 2.1–3.6). Consistent with this, in a univariate analysis, women who actually lived with their partner were 3.1 fold (95 % CI OR = 2.3–4.2) more likely to be part-time sex workers than those not living with their partners, or who were single (data not shown).

**Table 2:** Association between migrant type and economic dependence on sex work, work conditions and health contact among female sex workers in four sites in South Africa (N = 1636)

Variables	Non-migrant n = 240; A	Internal migrant n = 638; B	Cross-border migrant; n = 758; C	*P* value A vs C^a^	*P* value B vs C^a^
Economic dependence on sex work					
Part-time sex work, n/N (%)	40/213 (18.8 %)	150/606 (24.8 %)	256/723 (35.4 %)	<0.001	<0.001
Median amount charged with last client US$ (IQR, range), n	13.3 (13.3–24; 2.7–458.4), n = 233	13.3 (6.7–26.7; 1.5–466.7), n = 629	20.0 (10.7–40; 0.3–466.7), n = 754	0.01^b^	<0.001^b^
Unsafe working conditions^c^					
Police interaction, last year, n/N (%)	86/197 (43.6 %)	217/537 (40.4 %)	277/624 (44.4 %)	0.86	0.17
Positive^d^	2/197 (1.0 %)	2/537 (0.4 %)	10/624 (1.6 %)	0.55	0.04
Negative^e^	59/197 (30.0 %)	140/537 (26.1 %)	192/624 (30.7 %)	0.83	0.08
Nature of negative police interaction in last year, n/N (%)^f^					
Physical/sexual assault	9/197 (4.6 %)	24/537 (4.5 %)	24/624 (3.8 %)	0.65	0.60
Bribe	5/197 (2.5 %)	197/537 (3.2 %)	33/624 (5.2 %)	0.11	0.08
Immigration issues	1/197 (0.5 %)	1/537 (0.2 %)	34/624 (5.5 %)	0.003	<0.001
Arrest	40/197 (20.3 %)	96/537 (17.8 %)	109/624 (17.5 %)	0.37	0.86
Other	24/197 (12.2 %)	39/537 (7.3 %)	57/624 (9.1 %)	0.21	0.25
Contact with health services					
Received facility or community-based services in last month, n/N (%)	131/216 (60.7 %)	352/595 (59.2 %)	421/718 (58.6 %)	0.60	0.85
Condom-use with last client during penetrative intercourse, n/N (%)	217/230 (94.6 %)	558/626 (89.1 %)	677/747 (90.6 %)	0.08	0.36

1US$ = 7.5 South African Rand

^a^Chi square test unless indicated

^b^Mann-Whitney *U* test

^c^Post-coding free-text answers. Some participants gave insufficient information to classify interaction as positive or negative

^d^Police assistance with laying a complaint or warning a participant about potential danger

^e^Police violence, arrest, harassment, theft, bribery or fines

^f^Multiple-response question

**Table 3:** Multivariate analysis of factors associated with part-time sex work, negative police interaction and health care utilization among female sex workers in South Africa

Variable	Part-time sex work		Negative police interaction		Health care utilization	
	Univariate OR (95 % CI)	Multivariate OR (95 % CI)	Univariate OR (95 % CI)	Multivariate OR (95 % CI)	Univariate OR (95 % CI)	Multivariate OR (95 % CI)
Age						
18–24	1.0	1.0	1.0	–	1.0	1.0
25–30	1.14 (0.83–1.56)	1.38 (0.94–2.04)	1.20 (0.85–1.69)		1.26 (0.94–1.69)	1.00 (0.72–1.39)
30–35	1.11 (0.80–1.56)	1.62 (1.08–2.45)	1.38 (0.97–1.96)		0.89 (0.66–1.20)	0.82 (0.58–1.15)
35+	1.18 (0.85-1.65)	1.72 (1.11–2.68)	1.00 (0.69–1.45)		0.87 (0.64–1.18)	0.77 (0.54–1.11)
Site						
Cape Town	1.0	1.0	1.0	1.0	1.0	1.0
Hillbrow, Johannesburg	0.95 (0.71–1.28)	0.44 (0.29–0.67)	0.86 (0.63–1.17)	1.04 (0.68–1.57)	1.87 (1.39–2.50)	1.75 (1.21–2.52)
Rustenburg	0.65 (0.46–0.90)	0.38 (0.25–0.60)	0.10 (0.06–0.18)	0.06 (0.03–0.13)	0.43 (0.32–0.58)	0.48 (0.34–0.69)
Sandton, Johannesburg	1.18 (0.83–1.67)	0.54 (0.34–0.86)	2.12 (1.47–3.05)	1.82 (1.15–2.88)	0.81 (0.58–1.14)	0.82 (0.56–1.21)
Migration status						
Non-migrant	1.0	1.0	1.0	1.0	1.0	1.0
Internal migrant	1.42 (0.96–2.10)	1.47 (0.93–2.31)	0.82 (0.58–1.18)	0.88 (0.56–1.38)	0.94 (0.68–1.29)	0.65 (0.45–0.93)
Cross-border migrant	2.37 (1.63–3.45)	2.34 (1.47–3.71)	1.04 (0.73–1.47)	1.27 (0.80–2.02)	0.92 (0.67–1.25)	0.59 (0.40–0.86)
Education						
Primary incomplete	1.0	1.0	1.0	1.0	1.0	1.0
Completed primary	1.30 (0.94–1.79)	1.34 (0.89–2.01)	1.15 (0.83–1.60)	0.95 (0.61–1.46)	1.40 (1.06–1.85)	0.85 (0.62–1.18)
Completed secondary school	1.41 (0.98–2.02)	1.29 (0.82–2.02)	1.11 (0.76–1.61)	0.91 (0.56–1.48)	1.26 (0.92–1.74)	0.77 (0.53–1.11)
Some tertiary training	2.39 (1.46–3.91)	2.00 (1.12–3.59)	0.50 (0.27–0.92)	0.46 (0.22–0.94)	1.45 (0.90–2.33)	1.03 (0.61–1.74)
Number of dependants						
0	1.0	–	1.0	1.0	1.0	1.0
1–3	1.13 (0.73–1.76)		1.03 (0.66–1.61)	0.89 (0.52–1.55)	1.81 (1.25–2.64)	1.60 (1.04–2.44)
≥4 or more	1.78 (1.16–2.72)		1.20 (0.78–1.86)	0.78 (0.45–1.35)	2.79 (1.93–4.03)	2.09 (1.35–3.25)
Relationship status						
Permanent partner	1.0	1.0	1.0	–	1.0	1.0
Single	3.41 (2.71–4.30)	2.77 (2.13–3.60)	1.24 (0.97–1.58)		1.62 (1.30–2.01)	1.48 (1.16–1.89)
Main place solicits clients^a^						
Indoors	1.0	1.0	1.0	1.0	1.0	1.0
Outdoors	0.59 (0.45–0.78)	0.52 (0.37–0.74)	2.09 (1.58–2.76)	1.64 (1.15–2.36)	0.75 (0.59–0.96)	0.83 (0.62–1.10)
Combination of venues	1.14 (0.87–1.50)	1.04 (0.76–1.42)	1.60 (1.18–2.16)	1.35 (0.95–1.91)	0.95 (0.73–1.23)	1.04 (0.77–1.39)
Duration in sex work						
<1 years	1.0	1.0	1.0	1.0	1.0	–
1–5 years	0.84 (0.60–1.16)	0.71 (0.49–1.04)	1.93 (1.27–2.94)	2.15 (1.36–3.39)	0.99 (0.73–1.36)	
>5 years	0.70 (0.51–0.98)	0.63 (0.42–0.95)	1.65 (1.09–2.51)	2.83 (1.78–4.53)	0.81 (0.60–1.10)	

*OR* odds ratio, *CI* confidence interval

^a^Indoors includes working from brothels, bars or massage parlours; outdoors includes street-based sex workers

Cross-border migrants charged a median $7 more with their last client ($20), than internal migrants (*P* < 0.001) or non-migrants (*P* = 0.01). Median number of clients in the past week was 14 for cross-border and 15 for internal migrants, double the median number of clients of non-migrants (*P* < 0.001). Zimbabwean women had a considerably higher median number of clients per week (n = 18), than their counterparts from South African (n = 11) or elsewhere (n = 12; *P* ≤ 0.001). Among full-time sex workers only, non-migrants received the lowest weekly income at $126.70 (IQR = 65.3–280) compared to internal migrants’ $200 (IQR = 88–466.7) and the $233.33 (IQR = 116.7–554.6; *P* < 0.001) of cross-border migrants (data not shown).

### Unsafe Work Conditions

More than 40 % of participants had some contact with police in the past year, with almost a third having a negative experience. Occurrences were similar across study groups, including in multivariate analysis, though the nature of police interaction differed. Cross-border migrants had more experience of police bribes (5.2 %) or issues relating to immigration (5.5 %) than the internal migrants (3.2 and 0.2 %, respectively) and non-migrants (2.5 and 0.5 %, respectively). In Hillbrow, 9.8 % of cross-border sex workers had interacted with police on immigration, as opposed to 4.9 % in Sandton, 0.6 % in Rustenburg and 0 % in Cape Town. Sex workers in outdoor settings were 1.6 fold (AOR, 95 % CI = 1.2–2.4) more likely to have adverse interactions, than women in indoor settings. Also, negative police interaction was more than twice as likely among those in the industry for 1–5 years than those who had just started sex work (AOR = 2.2; 95 % CI = 1.4–3.4), and such encounters were almost three times as likely among those in the industry for more than 5 years (95 % CI = 1.8–4.5). FSWs in Rustenburg were much less likely to experience negative police interaction than those in Cape Town (AOR = 0.06; 95 % CI = 0.03–0.13), though levels in Sandton were 1.82-fold higher than the latter city (95 % CI AOR = 1.15–2.88).

### Health Care Utilization

Close to 60 % of participants in each group interacted with health service in the last month. However, in a sub-analysis of utilization in Cape Town, non-migrants had more contact than cross-border sex workers (72.8 vs. 50.0 %; *P* = 0.002), and 81.8 % of non-migrants had contact in the past month in Hillbrow versus 75.0 % of cross-border migrants (*P* = 0.38, data not shown). In multivariate analysis controlling for site and other confounders, cross-border migrants were less likely to access health care (AOR = 0.6; 95 % CI = 0.4–0.9; Table 3) than non-migrants. Health contact was considerably higher in Hillbrow than other sites. Non-migrants were more likely to use a condom during penetrative sex with last client (217/230; 94.6 %) than internal (558/626; 89.1 %; *P* = 0.02, data not shown) or cross-border migrants (677/747; 90.6 %; *P* = 0.08).

## Discussion

In this survey, nearly half of FSWs were cross-border migrants. Two-thirds of the cross-border sex workers in Hillbrow migrated from neighbouring Zimbabwe, mirroring the escalation in Zimbabwean migration to South Africa in search of improved livelihood opportunities following political and economic instability in Zimbabwe since the early 2000 s [21, 63].

Our data challenges prevailing assumptions that position cross-migrants as the most disempowered sub-group within the sex industry [15, 34]. Compared to their internal or non-migrant colleagues, cross-border sex workers in this study had spent less time in the industry, had additional income-generating activities, worked mostly in the relatively safer indoor venues, and were older when they made their sex work debut. Cross-border migrants were also better educated than internal or non-migrants, similar to other studies in South Africa [26, 28]. Finally, this population had a higher client number than non-migrants, and charged more per client than internal or non-migrants.

Surprisingly few differences were observed in police interaction amongst the migrant groups. More cross-border migrants reported police requesting a bribe, possibly reflecting police’s practice of extorting money or favours from cross-border migrants in relation to their status as non-nationals [49, 66]. Likely over time, police become familiar with sex workers in an area, explaining why interaction with police increases with duration in the industry.

Higher levels of contact with health services in Hillbrow could be attributed to the only sex work-specific clinic in South Africa operating there [42]. Overall, cross-border migrants had considerably less contact with health services than the other groups in multivariate analysis. Similarly, a study in Nairobi, Kenya found only 55 % of migrant FSWs had ever accessed a health facility for an HIV-test in comparison to 78 % of FSWs born in Kenya [24]. This may reflect an unwillingness of cross-border migrants to engage with public facilities due to fear of arrest in the case of an irregular legal status, or as a result of prior negative experiences [23, 47, 61], or as peer education services do not adequately reach this group. Migrant sex workers, compared to non-migrants, face greater discrimination and additional barriers to health, as well as social and legal services [53–55, 64]. Alternatively, it may point to the ‘healthy migrant effect’, where immigrants to a new community may on average be healthier on arrival than the host population [14, 30]. Regardless of the reason(s), strategies are required to ensure cross-border migrant sex workers can utilize health services, and in particular HIV and STI prevention and treatment services, when needed [31, 59, 60].

The study has several limitations. It used a non-random sampling design and includes only self-reported data. Surveys were, however, conducted by trained peer interviewers—many migrants themselves—which may have minimised social-desirability bias. Multiple comparisons were made between study groups, increasing the changes of spurious findings. Even though questionnaires were available in five of the most widely spoken languages, some cross-border migrants may not be conversant in these, precluding their participation. Research sites were purposively selected and may not be generalizable to other sex work settings within the country. The three outcome variables selected describe only a limited number of risk factors associated with sex worker ill health and several others should have been assessed. In particular, workplace safety encompasses several factors other than negative police contact, such as exploitative managers or controllers, a violent neighbourhood and no condom supplies within sex work venues [3, 65]. Also, additional factors such as irregular immigration status, ethnic or racial discrimination and ghettoised work conditions are pertinent to migrant sex workers, as shown elsewhere [37]. Similarly, there may be instances where women elected to be full-time sex workers because of its comparative higher earnings (not because of lack of alternatives) and they may make sufficient money to resist client overtures for unprotected sex.

In conclusion, our data illustrate the preponderance of migrants in sex work and the relative tenacity of cross-border migrants in South Africa. It illustrates the need for further sex work-specific health services, which specifically address health needs of migrant sex workers, especially around HIV/STI prevention. Such services should actively involve migrant sex workers in their design and planning, and as peer educators and outreach workers.
